# Preliminary data on *Pemphigus vulgaris *treatment by a proteomics-defined peptide: a case report

**DOI:** 10.1186/1479-5876-4-43

**Published:** 2006-10-24

**Authors:** Giovanni Angelini, Domenico Bonamonte, Alberta Lucchese, Gianfranco Favia, Rosario Serpico, Abraham Mittelman, Simone Simone, Animesh A Sinha, Darja Kanduc

**Affiliations:** 1 Department of Internal Medicine, Immunology and Infectious Diseases, Dermatology Section, University of Bari, Italy; 2 Department of Odontostomatology and Surgery, University of Bari, Italy; 3 Institute of Clinical Odontostomatology, 2^nd ^University of Naples, Italy; 4 Department of Medicine, New York Medical College, Valhalla, NY, USA; 5 Department of Biochemistry and Molecular Biology, University of Bari, Italy; 6 Division of Dermatology and Cutaneous Sciences, Michigan State University, East Lansing, USA

## Abstract

**Background:**

Although described by Hippocrates in 400 B.C., pemphigus disease still needs a safe therapeutical approach, given that the currently used therapies (i.e. corticosteroids and immunosuppressive drugs) often provoke collateral effects. Here we present preliminary data on the possible use of a proteomics derived desmoglein peptide which appears promising in halting disease progression without adverse effects.

**Methods:**

The low-similarity Dsg3_49–60_REWVKFAKPCRE peptide was topically applied for 1 wk onto a lesion in a patient with a late-stage Pemphigus vulgaris (PV) complicated by diabetes and cataract disease. The peptide was applied as an adjuvant in combination with the standard corticosteroid-based immunosuppressive treatment.

**Results:**

After 1 wk, the treated PV eroded lesion appeared dimensionally reduced and with an increased rate of re-epithelization when compared to adjacent non-treated lesions. Short-term benefits were: decrease of anti-Dsg antibody titer and reduction of the corticosteroid dosage. Long-term benefits: after two years following the unique 1-wk topical treatment, the decrease of anti-Dsg antibody titer persists. The patient is still at the low cortisone dosage. Adverse effects: no adverse effect could be monitored.

**Conclusion:**

With the limits inherent to any preliminary study, this case report indicates that topical treatment with Dsg3_49–60_REWVKFAKPCRE peptide may represent a feasible first step in the search for a simple, effective and safe treatment of PV.

## Background

*Pemphigus vulgaris *(PV) is a rare, but severe immune-mediated blistering skin disease mediated by autoantibodies which bind to the cell surface of keratinocytes. The first recorded instance of Pemphigus disease was by Hippocrates (460–370 BC) who described pemphigoid fever as "*pemphigodes pyertoi*." Galen (131–201 AD) named a pustular disease of the mouth as "*febris pemphigodes*." In 1791, Wichmann used the term "pemphigus" to indicate a pathology characterized by flaccid bullae and painful oral ulcerations. In 1964 Beutner and Jordon reported autoantibodies in the sera of pemphigus patients, reactive with an "intercellular substance" of skin and mucosa, by using indirect immunofluorescence [1,2]. Eventually, in 1990 Amagai, Klaus-Kovtun and Stanley identified the "intercellular substance" as desmoglein-3, a 130-kDa desmosomal adhesion molecule [3]. Today the pathogenicity of anti-Dsg3 autoantibodies is a datum of fact since transfer of patient derived anti-Dsg3 serum IgG antibodies into mice induces a bullous skin disease resembling PV [4].

Histopathologically, PV is characterized by suprabasal intraepidermal bullae with acantholysis and inflammatory infiltrate of eosinophils. Immunopathologically, IgG and C3 deposits are found in intercellular/cell surface areas in skin lesions. Typically, Nikolsky's sign is present in this disease: sheetlike removal of skin by gentle pushing with a finger [5,6]. Although histologically well characterized, the course of the pemphigus pathological events and the specific pathway of the blistering process is not fully understood. In parallel, the molecular basis and the biochemical events of the pemphigus pathology remain to be clearly defined.

Therapeutically, PV treatments include corticosteroids, immunosuppressive drugs (azathioprine, cyclophosphamide, cyclosporine, and methotrexate), anti-inflammatory agents (gold, dapsone, tetracycline and nicotinamide) [5-12], plasmapheresis [13] and, more recently, intravenous immunoglobulins [14-17] and cholinergic agonists [18]. The final goal of these treatments is to reduce inflammation and/or production of the pathogenic autoreactive antibodies. There are several limitations that make current treatment protocols less than ideal: 1) no single therapy, other than high-dose steroid administration, has been reported resolutive so far; 2) prolonged immunosuppression may be associated with severe side effects, including an enhanced susceptibility to opportunistic infections; 3) the efficacy of high-dose steroid administration is transient, and relapses are the rule as soon as the steroid treatment is discontinued. Moreover, the side-effects of corticosteroid treatment are numerous and heavy, one example for all being represented by steroid-induced diabetes [19-21].

In such a context, the need for the development of alternative, effective and safe treatments for PV is unquestionable and mandatory. In our labs, we are testing the possibility of applying peptide-immunotherapy targeted to specific low-similarity protein segments, thereby treating the disease without the risk of collateral cross reactions [22-31]. Accordingly, in the present approach to PV peptide immunotherapy we have used a linear low-similarity segment of the protein autoantigen associated to PV, desmoglein-3 (Dsg3) amino acid 59–60 corresponding to the sequence REWVKFAKPCRE [32,33]. The low-similarity peptide was defined using a proteome-base computer-assisted algorithm network in order to identify Dsg3 peptide fragments potentially able to interfere with and/or stop the PV pathological event chain and, at the same time, eliminate possible collateral effects due to cross reactions. Following a series of *in vitro *and animal experiments [32-34], our studies have progressively focused on the Dsg3_49–60_REWVKFAKPCRE peptide sequence that 1) is uniquely expressed in Dsg3 and, consequently, cannot induce/provoke collateral secondary autoimmune cross-reactions [22-34]; 2) is hosted in a Dsg3 domain involved in the intramolecular epitope spreading characterizing the progression of PV from mucous to muco-cutaneous stage [35]; 3) does not produce pathogenic antibodies [33].

Here we describe a case report illustrating the potential therapeutic use of the computer-designed Dsg3_49–60_REWVKFAKPCRE peptide in PV.

## Materials and methods

### Peptide description

The EC1/EC2 Dsg3_49–60_REWVKFAKPCRE peptide was synthesized using standard Fmoc (N-(9-fluorenyl)methoxycarbonyl) solid phase peptide synthesis. Peptide purity (>95%) was controlled by HPLC, and the molecular mass of purified peptide confirmed by fast atomic bombardment mass spectrometry.

### Treatment design

Dsg3_49–60_REWVKFAKPCRE peptide was administered by topical route. A cream formulation of pure vaseline containing 0.1% Dsg3_49–60_REWVKFAKPCRE peptide was prepared under sterile conditions. Peptide immunotherapy was carried out on a late stage PV patient by using the following provisos: 1) no canonical medical treatment was discontinued and/or modified; 2) peptide immunotherapy was administered under form of topical cream; 3) the topical peptide treatment was temporally limited to one week; 4) the topical peptide treatment was locally limited to only one out of the patient's multiple erosive skin lesions; 5) the patient was hospitalized and clinically monitored in order to discontinue the treatment at the least sign of discomfort and, afterwards, remains under control for nearly two years past the topical treatment; 6) preliminarly, the possibility that the anti-Dsg3_49–60_REWVKFAKPCRE peptide might be pathogenic (and, consequently, harmful to the patient) had been excluded [33].

### Patient's history and treatment

The 1-wk topical application of the peptide cream was conducted at the Department of Dermatology, Faculty of Medicine, University of Bari in agreement to the Helsinki declaration, with patient's detailed informed consent, and applying the guidelines regulating biomedical research involving human subjects.

The patient (56-year-old, 56 kg) presented with 4-year history of mucocutaneous PV, with multiple relapses, and numerous hospitalization, during which he had been under care at the Dermatology Clinic of the Faculty of Medicine, University of Bari. The diagnosis of pemphigus had been confirmed at the onset by excisional biopsy and histology, Tzanck's cytodiagnostic method, direct immunofluorescene (DIF) and indirect immunofluorescene (IIF). The latter investigations revealed acantholytic phenomena linked to a high titer of circulating IgG AAbs (titer ≥ 1:400).

Starting in 2001, the patient had been treated with systemic corticosteroids (100 mgs/die deltacortene) plus azathioprine (100 mgs/die) and cyclosporine (300 mgs/die). When corticosteroid dosage was progressively downscaled, disease recurrence occurred at doses lower than 30 mgs/die). Because of collateral treatment effects (diabetes mellitus, arterial hypertension, corneal ulcers), the patient also underwent several sessions of plasmapheresis which led to temporary clinical improvement and reduction in the anti-Dsg3 AAb titer.

On May 2004, the subject was hospitalized because of a severe relapse. At this time the patient presented vast areas of disepithelization, especially on the back, axillary and inguino-scrotal folds plus numerous erosions in the oral mucosa. Nikolsky's sign was positive. Blood test revealed hypochromic anemia, mild hypoproteinemia and hypocalcemia as well as type II diabetes mellitus. The patient had also signs of infection of the urinary tract.

Given the severe clinical picture, the hospitalized patient was started on aggressive corticosteroid therapy (as already administered in the past), administered in 1 g sodium hydrocortisone hemisuccinate/die by i.v. route for three consecutive days. Corticosteroids were subsequently scaled down to 100 mg/die prednisone, and further lowered to topical application of clobetasol propionate cram twice a day. After 10 days, IFI decreased by 50% (AAb titer 1:200).

**Figure 1:**
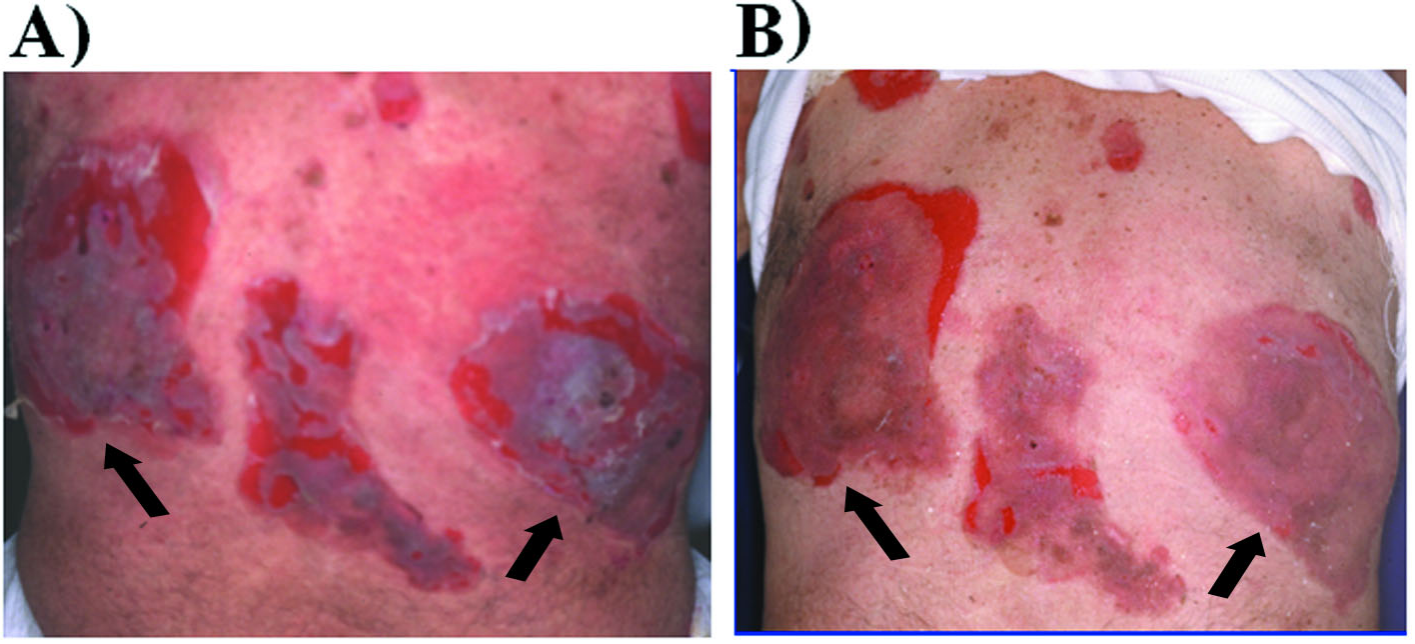
Clinical results following Dsg3 peptide-cream topical application. A) Patient's lesions before the peptide treatment. Arrows indicate the area selected to be treated with clobetasol propionate (on the left) or the peptide cream (on the right). B) After 1 wk of clobetasol propionate treatment (on the left) or peptide cream application (on the right).

At this time, the Dsg3_49–60_REWVKFAKPCRE peptide cream (0.1% peptide in pure vaseline) treatment was started. Specifically, two symmetrical areas hosting PV lesions (disepithelization, erosions and evident inflammation) were devised on the back (see Fig. 1A), and the peptide cream was applied twice a day to the lesion on the right, while the symmetrical eroded lesion on the left received topical application of clobetasol propionate cream. The Dsg3 peptide-cream treatment was continued for 7-days, during which the peptide cream was applied topically on the right back lesion, twice daily. Afterwards the patient remained hospitalized and under observation for a further 10 days.

The patient was discharged from hospital with a reduced corticosteroid prescription (50 mgs deltacortene/die), further reduced to 20 mgs/day (see Table 1) after 5 mo. Today the patient is still under control and, at nearly 2 years from that unique topical treatment, has not undergone any relapse, remains at a regimen of 20 mgs deltacortene/day and presents a clinical picture that can be defined as under control (see data in Table 1).

**Table 1:** Corticosteroid dosage and anti-dsg3 AAb level. Patient's follow-up.

Time	Deltacortene (mgs/die)	IIF Titer
Prior to May 2004	100	1:400
May 2004*	50	1:200
November 2004	20	1:100
February 2005	20	1:100
March 2006	20	1:100

* Topical peptide treatment given.

## Results

### Scientific rationale of the Dsg3_49–60_REWVKFAKPCRE-based treatment

Peptide immunotherapy has enormous potential to slow down the progression of malignant, autoimmune, allergic and infectious diseases [39-50]. But the consideration of safety of the treatment is paramount: in recognising the potential effectiveness of this therapy, it cannot be stressed enough that great care must be taken when attempting to suppress/modify any autoimmune diseases using peptide immunotherapy. In fact, peptides are able to cause dangerous autoimmune responses if they evoke cross-reactions with other normal housekeeping proteins and/or are pathogenic by themselves [51,52].

Given these caveats, the Dsg3_49–60_REWVKFAKPCRE peptide appeared safe and promising of effectiveness since we have already demonstrated that the linear Dsg3_49–60_REWVKFAKPCRE peptide sequence corresponds to a motif having a low degree of sequence similarity to host's proteome. Theoretically, this would nullify the possibility of cross-reactions between anti-Dsg3_49–60 _REWVKFAKPCRE antibodies and other unrelated host's proteins hosting the Dsg3 motif under examination. The possibility that the linear Dsg3_49–60_REWVKFAKPCRE peptide might be pathogenic is further to be unlikely by the fact that the Dsg_1–87 _portion of the Dsg protein appears not to be involved in the induction of PV. AAbs from patients at the earlier stage recognize an epitope located at Dsg3_87–566 _but not an epitope located at the first 87 amino acids. It is noted, however, that the autoimmune humoral response of patients in the later stage of disease can be targeted towards an epitope located in the first 87 amino acids of Dsg3 [33]. Moreover, a recent work from our labs has demonstrated that IgG AAbs elicited against the Dsg3_49–60_REWVKFAKPCRE peptide do not have pathogenic potential, providing experimental support to contradict the possibility that the peptide administration might generate pathogenic anti-Dsg3 antibodies capable of exhacerbating pemphigus pathology.

### Clinical results following peptide-cream topical application

From the very first days of application, the peptide-treated lesion showed a higher rate of re-epithelization compared to the clobetasol propionate-treated area. Peptide treatment lasted 7-days. As illustrated in Fig. 1, the area treated with the peptide cream shows resolution of the dermatitis, in contrast to the contralateral lesion which still presents signs of disepithelization, mostly at the periphery. Careful clinical examination of the treated lesion put into evidence the following results when compared to non-treated lesions: a) almost immediate disappearance of the circular extra-lesion inflammation redness; b) reduction of lesion size; c) increase of the re-epithelization rate c) reduction of the erosions present in the lesion, both numerically and as area extension (Fig. 1B). There was no evidence of any adverse effect. The patient expressed well-being and highlighted the higher ease he had in moving and lying on the body part corresponding to the treated lesion.

Subsequent monitoring post-peptide cream application revealed a progressive decrease of anti-Dsg3 AAb titer, as detailed in Table 1. It is noteworthy that about 2 years after that unique peptide-therapy topical treatment, anti-Dsg3 titer still is low (1:100). As importantly, no relapse has been monitored notwithstanding the patient remains at a low regimen of corticosteroids (20 mgs/die) (Table 1).

## Discussion

The "peptide vaccine" concept is based on the identification and chemical synthesis of B cell and/or T cell epitopes that are immunodominant and may induce specific immune functions (neutralization, killing, help). Peptide mimics, cyclic peptides, branched peptides, peptomers (cross-linked peptide polymers) and other complex multimeric structures, as well as peptides conjugated to other molecules (see lipopeptides), have been developed and in a few instances even tested in the attempt of identify effective peptide sequence/formulations in immunotherapeutical approaches to cancer and autoimmune disease [36-48]. The simplest peptide form in vaccine formulations is represented by linear polymers of ~8–24 amino acids. Linear amino acids sequences offer a more reductionist approach to evaluate effectiveness and results both in vivo and in vitro biomedical research [22,23]. Most importantly, preliminarily to scoring for effectiveness, a peptide vaccine must be proved to be safe and void of side effects as a first priority. Unfortunately, several peptide vaccine immunotherapeutical approaches to date have shown a concomitant sequela of harmful, sometimes lethal, autoimmune reactions due to cross reactions with not only the disease-associated-protein but also other unrelated housekeeping proteins [49-52] possibly sharing the same peptide sequence [24].

As regards PV, the HLA selective presentation of DSG3 peptides in PV has been thoroughly analyzed, starting from the premise that peptide presentation to T cells may be important in the initiation or progression of autoimmune diseases. Based on the DRB1*0402 binding motif, seven DSG3 candidate peptides were identified by Wuchepfennig et al. [53]. Accordingly, previously identified stimulatory Dsg3 peptides were docked into the binding groove of constructed atomic models of ten PV associated, non-associated and protective alleles, in order to analyze the structural aspects of allele-specific binding [54]. However, to our knowledge, none of the HLA-binding DSG3 peptides studied so far has produced possible therapeutical applications. Rather, additional studies in PV patients and healthy subjects have further confirmed that T cell recognition of Dsg3 peptides is tightly restricted by distinct HLA class II alleles, but, also, have demonstrated that T cell recognition of distinct Dsg3 peptides is independent of the development of *Pemphigus vulgaris *[55].

Our strategy was to use an *in silico *technology proteomics-based platform to identify epitopic peptide sequence(s) from disease-associated-antigens that share no similarity with the host proteome [22-34]. By identifying low/no similarity level peptide sequences, our proteomic approach offers an effective and safe tool to specifically identify motifs uniquely expressed in the disease-associated-protein, in an effort to avoid the possibility of unwanted cross-reactions.

In applying this scientific rationale to the *Pemphigus vulgaris *associated antigen, Dsg3, we have identified the Dsg3_49–60_REWVKFAKPCRE peptide sequence as a fragment with low similarity to human proteome [32-34]. Based on this property, Dsg3_49–60_REWVKFAKPCRE peptide is postulated to represent a safe and secure base for immunotherapy of pemphigus. Consequently we: 1) formulated a topical Dsg3_49–60_REWVKFAKPCRE peptide cream and 2) elaborated a likewise safe and secure application protocol consisting of a 1 wk application time over a limited application area. This report represents an exemplar of the cautions and provisos that have to be observed for peptide immunotherapy. *De facto*, the Dsg3_49–60_REWVKFAKPCRE peptide topical application has given short- and long-term positive results in terms of lesion healing and the patient's well being, with an absence of any adverse effect.

In conclusion, topical treatment with Dsg3_49–60_REWVKFAKPCRE peptide deserves further clinical investigation in order to open the way towards a careful, cautious and safe long-term PV immunotherapy by topically applied, or even parenterally administered, Dsg3_49–60_REWVKFAKPCRE peptide. More in general, this report promotes the concept that peptides with low level of similarity to the host's proteome might safely be used in immunotherapy of autoimmune diseases.

## Authors' contributions

GA and DB carried out the clinical treatment and the patient's follow up; AL, GF, RS, AM, SS, AAS participated in the design of the study, and analyzed and controlled the data; DK conceived the study, and participated in its design and coordination. All authors read and approved the final manuscript.
